# *In Silico* Genome-Wide Analysis Reveals the Potential Links Between Core Genome of *Acidithiobacillus thiooxidans* and Its Autotrophic Lifestyle

**DOI:** 10.3389/fmicb.2018.01255

**Published:** 2018-06-08

**Authors:** Xian Zhang, Zhenghua Liu, Guanyun Wei, Fei Yang, Xueduan Liu

**Affiliations:** ^1^Department of Occupational and Environmental Health, Xiangya School of Public Health, Central South University, Changsha, China; ^2^School of Minerals Processing and Bioengineering, Central South University, Changsha, China; ^3^College of Life Science, Nanjing Normal University, Nanjing, China; ^4^Key Laboratory of Biometallurgy of Ministry of Education, Central South University, Changsha, China

**Keywords:** *Acidithiobacillus thiooxidans*, core genome, mathematical modeling, bacterial lifestyle, metabolic profiles

## Abstract

The coinage “pan-genome” was first introduced dating back to 2005, and was used to elaborate the entire gene repertoire of any given species. Core genome consists of genes shared by all bacterial strains studied and is considered to encode essential functions associated with species’ basic biology and phenotypes, yet its relatedness with bacterial lifestyle of the species remains elusive. We performed the pan-genome analysis of sulfur-oxidizing acidophile *Acidithiobacillus thiooxidans* as a case study to highlight species’ core genome and its relevance with autotrophic lifestyle of bacterial species. The mathematical modeling based on bacterial genomes of *A. thiooxidans* species, including a novel strain ZBY isolated from Zambian copper mine plus eight other recognized strains, was attempted to extrapolate the expansion of its pan-genome, suggesting that *A. thiooxidans* pan-genome is closed. Further investigation revealed a common set of genes, many of which were assigned to metabolic profiles, notably with respect to energy metabolism, amino acid metabolism, and carbohydrate metabolism. The predicted metabolic profiles of *A. thiooxidans* were characterized by the fixation of inorganic carbon, assimilation of nitrogen compounds, and aerobic oxidation of various sulfur species. Notably, several hydrogenase (H_2_ase)-like genes dispersed in core genome might represent the novel classes due to the potential functional disparities, despite being closely related homologous genes that code for H_2_ase. Overall, the findings shed light on the distinguishing features of *A. thiooxidans* genomes on a global scale, and extend the understanding of its conserved core genome pertaining to autotrophic lifestyle.

## Introduction

The development of next-generation sequencing technologies has engendered the public databases inundated with plentiful sequenced genomes (Vernikos et al., 2015). Using the gene content of microbial genomes, large-scale comparative genomics has advanced our understanding of extensive intra-species diversity (Pallen and Wren, 2007; Zhang et al., 2016a). To address the question of how many genomes were needed to fully characterize a bacterial species, the best approximation was to introduce the concept of pan-genome (“pan,” derived from Greek word “παν,” meaning “whole”; Medini et al., 2005; Tettelin et al., 2005), which was first coined to define the global gene repertoire across all strains of individual species. Core genome consists of genes shared by all strains of any given species, and probably encodes functions necessary for its fundamental biological processes and phenotypes (Medini et al., 2005; Tettelin et al., 2008). And the remaining is dispensable genome that contains genes present in some but not all the strains studied and strain-specific genes, and probably contributes to the species’ diversity (Tettelin et al., 2008). Pan-genome analyses provide a framework not only to estimate the genomic diversity of a species using the datasets at hand, but also to predict, via extrapolation, the extension of bacterial pan-genome (Vernikos et al., 2015). Mathematical modeling based on bacterial pan-genomes indicated that in the species with an open pan-genome, novel genes would be added to the gene repertoire of a species with new genomes sequenced. In the species having the closed pan-genome, however, novel genomes sequenced might not provide additional new genes to expand the pan-genome (Vernikos et al., 2015). An open pan-genome hints that species has multiple ways of exchanging genetic material to extend the microbial gene pool. For a closed pan-genome, in contrast, species’ genome is more conserved due to a low capacity to acquire alien genes (Medini et al., 2005; Tettelin et al., 2005), indicating a more genome stability. And in fact, in both open and closed pan-genome, the essence of a species pertains to core genome, which is responsible for the basic biology and major phenotypic traits. Thus, it is of interest to investigate the genetic attributes of core genome that encodes for all possible lifestyles.

Members of the genus *Acidithiobacillus* (Kelly and Wood, 2000) are Gram-negative and rod-shaped bacteria, which are affiliated to the class *Acidithiobacillia* (Williams and Kelly, 2013). As of 2018, considerable efforts have been made to delineate several recognized species belonging to *Acidithiobacillus* genus: *Acidithiobacillus thiooxidans* (Waksman and Joffe, 1922), *Acidithiobacillus ferrooxidans* (Temple and Colmer, 1951), *Acidithiobacillus caldus* (Hallberg and Lindström, 1994), *Acidithiobacillus. albertensis* (Kelly and Wood, 2000), *Acidithiobacillus ferrivorans* (Hallberg et al., 2010), *Acidithiobacillus ferridurans* (Hedrich and Johnson, 2013a), and *Acidithiobacillus ferriphilus* (Falagán and Johnson, 2016). Very recently, a comprehensive phylogenetic analysis has been performed to elucidate the hierarchical relationships among an extensive set of *Acidithiobacillus* strains using molecular systematics approaches (Nuñez et al., 2017). It has been widely acknowledged that these microorganisms ubiquitously occur in both pristine ecological niches (e.g., acid rock drainage and sulfur springs) and acidic settings of anthropogenic origins (e.g., acid mine drainage and bioleaching operations; Valdés et al., 2008; You et al., 2011; Bonnefoy and Holmes, 2012; Chen et al., 2013; Talla et al., 2014; Yin et al., 2014a; Nuñez et al., 2017). Except for the scientific merits as model extreme acidophiles, the consortium of *Acidithiobacillus* isolates has been successfully exploited in the bio-hydrometallurgical operations for metal extraction from sulfur-bearing minerals (Johnson, 2014; Nuñez et al., 2016). Due to their capabilities of dissimilatory oxidation of elemental sulfur and a wide range of reduced inorganic sulfur compounds (RISCs), *Acidithiobacillus* species are believed to play a crucial role in the biogeochemical cycle of sulfur in the living habitats (Zhang et al., 2016c).

*Acidithiobacillus thiooxidans* (formerly *Thiobacillus thiooxidans*), a validated species of *Acidithiobacillus* genus, has been traditionally studied dating back to 1920s (Waksman and Joffe, 1922). It is a sulfide-oxidizing autotroph, which obtains energy derived from sulfur oxidation to support its autotrophic growth. In the last decade, numerous studies on sulfur oxidation of *A. thiooxidans* have been conducted mainly based on genome-wide analyses (Valdes et al., 2011; Travisany et al., 2014; Yin et al., 2014a,b; Zhang et al., 2015). Also, stoichiometric modeling has been attempted to construct the potential metabolic pathway of sulfur oxidation (Bobadilla Fazzini et al., 2013). In contrast to members of *A. ferrooxidans* and *A. ferrivorans*, *A. thiooxidans* isolates have no ability to aerobically oxidize the ferrous iron. In addition, hydrogen utilization was experimentally observed in several *Acidithiobacillus* organisms including *A. ferrooxidans*, *A. ferridurans*, and *A. caldus*, but not in *A. thiooxidans* and *A. ferrivorans* (Hedrich and Johnson, 2013b). Much of recent efforts have expanded the scope of genetic traits and bioenergetic pathways within *A. thiooxidans* strains.

In this study, nine draft genomes of *A. thiooxidans* strains (Valdes et al., 2011; Travisany et al., 2014; Yin et al., 2014b; Zhang et al., 2016a) were included. The geographic origins of these nine strains were previously reported in our earlier study (Zhang et al., 2016a), such as copper mine, Kimmeridge clay, and coal heap drainage worldwide. Comparisons of *A. thiooxidans* genomes have been made to gain a deeper appreciation of hereditary variation of *A. thiooxidans* isolates (Travisany et al., 2014; Zhang et al., 2016a). However, relatively few comparative analyses have focused on its conserved genome potentially related to the autotrophic lifestyle. Here a novel strain ZBY isolated from Zambian copper mine tailings was phylogenetically affiliated to *A. thiooxidans* species, and its genome was released to the GenBank database. Using the newly sequenced genome plus other recognized genomes of *A. thiooxidans* strains, pan-genome analysis presents the elucidation of their gene repertoire, metabolic profiles, and functional features. This work provides a coherent picture of genomic traits of *A. thiooxidans* isolates, and sheds light on the potential relevance between a large conserved common genes and autotrophic lifestyle of *A. thiooxidans* species.

## Materials and Methods

### Sample Collection, Strain Isolation, and Bacterial Culture

Samples were collected from bioleaching heaps at Chambishi copper mine in Zambia. Mineral samples were repeatedly flushed with distilled water (pH 2.0), and the solution was then filtered using a 0.22-μm pore-size filter membrane as previously described (Zhang et al., 2016d). Gradient dilution was implemented to isolate the targeted microorganisms according to their respective growth conditions. Before inoculation, elemental sulfur (boiling sterilized, 10 g/L) was added in liquid 9K basic culture medium. Bacterial strain was grown at 30°C in liquid medium on a shaking table at 170 rpm, as previously described by Yin et al. (2014a). After sequential dilution, pure isolate, designated ZBY, was aerobically cultivated at optimized conditions as above.

### Genomic DNA Extraction, Genome Sequencing, and Assembly

We collected bacterial cells at the stationary phase by centrifugation (12,000 g) for 20 min at 4°C. Total genomic DNA was extracted using TIANamp Bacteria DNA Kit (TIANGEN, Beijing, China), and the DNA quality was then assessed using a NanoDrop^®^ ND-1000 spectrophotometer (NanoDrop, Wilmington, DE, United States). The Illumina MiSeq platform (Illumina, CA, United States) was applied for whole-genome sequencing. We constructed the shotgun library with an average DNA insert size of 300 bp, which was used for 2 × 150 bp paired-end sequencing. After sequencing, all raw reads were screened using NGS QC Toolkit v2.3.1 (Patel and Jain, 2012) with the following parameters: cut-off read length for high-quality (HQ), 70%; cut-off quality score, 20. After quality control, the HQ sequences were *de novo* assembled using the Velvet program (Zerbino and Birney, 2008) with various k-mers. The best assembly was then used to generate unigene sequences by the iAssembler program (Zheng et al., 2011). GC content, N50, and N90 sequence length of genome assembly were calculated using the script “N50Stat.pl” within NGS QC Toolkit, and its completeness was evaluated using the CheckM program (Parks et al., 2015).

### Analysis of Phylogeny and Taxonomy

In order to establish how this strain was related to other *Acidithiobacillus* strains, we used the 16S rRNA sequences to construct a phylogeny. High sequence identities (≥97%) between newly sequenced strain and other recognized *Acidithiobacillus* strains preliminarily suggested their phylogenetic relationships. Accordingly, 16S rRNA gene sequences of *Acidithiobacillus* spp. deposited in GenBank database were acquired from individual genomes according to the approach as above. Multiple sequence alignment based on 16S rRNA sequences from various strains including newly sequenced strain was performed using a ClustalX v1.81. The ClustalX result was then used to construct the maximum likelihood tree implementing MEGA v5.05 (Tamura et al., 2011). Herein, the Tamura–Nei model of nucleotide substitution was applied for phylogenetic reconstruction. To evaluate clade support, we constructed a bootstrap analysis with 1,000 replicates. Furthermore, a genome-based phylogeny of *Acidithiobacillus* spp. was constructed using the online platform CVTree3 with K-tuple length 12 (Zuo and Hao, 2015). Here *Leptospirillum ferrooxidans* C2-3 was included as an out-group.

Comparisons of average nucleotide identity (ANI) based on BLAST algorithm (ANIb; Goris et al., 2007) and MUMmer algorithm (ANIm; Kurtz et al., 2004), as well as tetranucleotide frequency correlation coefficient (TETRA, Teeling et al., 2004) were further conducted using the software JSpecies v1.2.1 (Richter and Rossellómóra, 2009) to infer the phylogenetic relationships between pairs of bacterial genomes. For ANI calculation, default parameters were applied as follows: sequence identity cut-off, 30%; alignment cut-off, 70%; and query length, 1,020 bp. General features of the selected genomes are shown in Supplementary Table S1. A software HemI (Deng et al., 2014) was used to visualize the calculation results.

### Pan-Genome Analysis

In our study, nine of *A. thiooxidans* strains including new sequenced strain were selected for pan-genome analysis (Supplementary Table S2). Draft genome of ATCC 19377 was excluded in this part due to the low sequencing depth. We performed the BLASTP all-versus-all comparisons of entire amino acid sequences between pairs of *A. thiooxidans* strains. BLAST results with a tabular format (m8) were subsequently used to identify the orthologs among all of *A. thiooxidans* strains using the Pan-genome Ortholog Clustering Tool (PanOCT) v3.18 program (Fouts et al., 2012) with *E*-value cut-off of 1e^-5^, sequence identity cut-off of 50%, and match length cut-off of 65 bp. In this procedure, mobile genetic elements were removed from the datasets prior to the determination of orthologous genes as described previously (Snipen and Ussery, 2010). The results were then manually inspected and corrected.

### Mathematical Modeling of *A. thiooxidans* Pan-Genome

The sizes of core genome and novel genes of *A. thiooxidans* species depend on how many genomes are taken into consideration. To extrapolate the sizes of core-genome and pan-genome, the number of core and strain-specific genes were used to fit the exponential decaying curve *F_c_* = 𝜀*_c_*exp(-*n*/τ*_c_*) + Ω and *F_s_* = 𝜀*_s_*exp(-*n*/τ*_s_*) + *tg*(𝜃), respectively. In this formula, *n* is the number of strains and *𝜀_c_*, *𝜀_s_*, *τ_c_*, *τ_s_*, Ω, and *tg*(𝜃) are fitting parameters. Ω represents the size of core-genome and *tg*(𝜃) is the growth rate of pan-genome when *n* →∞ (Tettelin et al., 2005). Subsequently, we implemented the power law *P_s_* = κ*n*^-α^ (Tettelin et al., 2008) to determine whether the *A. thiooxidans* pan-genome is open (α ≤ 1) or closed (α > 1). Here, κ and α are fitting parameters. To evaluate the normality of the number of common and new genes, normality test was carried out by function “shapiro.test” in the package R v3.3.3 (R Core Team, 2000). In consideration of the data non-normality (Supplementary Figure S1), medians were used to estimate the average number of common and new genes when the *n*th genome was added. Parameter fitting were performed by using function “nls” with least-squares algorithm. The fitting results were summarized by function “summary.” Visualization for fitting curves were carried out by “ggplot2” package.

### Structural and Functional Analyses of Bacterial Genomes

NCBI prokaryotic genome annotation pipeline was applied to gene prediction and functional annotation of bacterial genome. Protein-coding sequences (CDSs) assigned to core genome and dispensable genome within strain ZBY were extracted using in-house script, and aligned against the specialized database, such as the extended Clusters of Orthologous Groups (COG; Franceschini et al., 2013); the results were screened according to the highest hit coverage value. Insertion sequences (ISs) and transposases were detected using the online platform ISfinder with an *E*-value cutoff set to 1e^-5^ (Siguier et al., 2006). The tRNAscan-SE program (Lowe and Eddy, 1997) was used for the identification of tRNA. Finally, BLASTN-based whole genome comparison was performed and visualized using the Circos software (Krzywinski et al., 2009) to exhibit the architecture and gene repertoire of *A. thiooxidans* pan-genome.

KEGG Automatic Annotation Server (KAAS; Moriya et al., 2007) was employed to infer the predicted metabolic profiles of core genome by sequence alignment against the manually curated KEGG GENES database. We gathered the amino acid sequences of interest for further analyses. Multiple sequence alignment was performed using the software DNAMAN v7.0.2, and phylogenetic tree based on the targeted proteins from various species was constructed using the MEGA v5.05. Additionally, pairwise comparisons of putative gene clusters within *A. thiooxidans* genomes were visualized using the software EasyFig v2.1 (Sullivan et al., 2011).

### Availability of Supporting Data

The datasets supporting the findings of this study are available in NCBI repository. The Whole Genome Shotgun project regarding the newly sequenced strain ZBY has been deposited at DDBJ/EMBL/GenBank under the accession number LZYI00000000. The version described in this paper is version LZYI01000000. All additional datasets generated and/or analyzed during the current study are included within the additional files of this article.

## Results

### General Genome Features of New Sequenced ZBY

HQ reads generated from quality control were used to genome assembly. As a result, the draft genome contains 3,793,418 base pairs (bps) distributed in 277 contigs and its GC content is 53.17%. The lengths of maximum and minimum contigs are 155,963 and 225 bp, respectively. Genome completeness evaluated by CheckM indicated that the genome assembly was near complete (99.34%, **Table 1**). Draft genome of novel strain ZBY was predicted to comprise 3,713 CDSs, 117 tRNA that cover all the 20 amino acids, and 134 ISs. Further alignment against the NCBI-*nr* database revealed that only 2,029 (54.65%) CDSs were assigned to putative proteins with known functions, while 1,684 (45.35%) CDSs were annotated as hypothetical proteins. Functional analysis based on COG classification showed that there were a total of 2,743 (73.88%) significant BLAST hits, in which 2,741 (73.82%) CDSs were assigned to the COG categories. The four most abundant functional categories within the ZBY genome were “replication, recombination, and repair (L),” “cell wall/membrane/envelope biogenesis (M),” “energy production and conversion (C),” and “amino acid transport and metabolism (E)” (Supplementary Table S3). Furthermore, COG annotation suggested that a plenty of poorly characterized sequences (20.74%) were dispersed in the draft genome of strain ZBY, making the potential roles unclear.

**Table 1:** General features of draft genome of newly sequenced strain ZBY.

Property	Value
Coverage	91×
Number of contigs	277
Total bases	3,793,418
Completeness (%)	99.34
Min sequence length (bp)	225
Max sequence length (bp)	155,963
N50 length (bp)	32,632
N90 length (bp)	6,269
GC content (%)	53.17
Total number of CDSs	3,713
tRNAs	117
IS elements	134
CDSs with known function	2,029 (54.65%)
Hypothetical proteins	1,684 (45.35%)
CDSs with COG hits	2,743 (73.88%)
CDSs with COGs	2,741 (73.82%)

### Phylogeny and Taxonomy of Novel Strain

**FIGURE 1:**
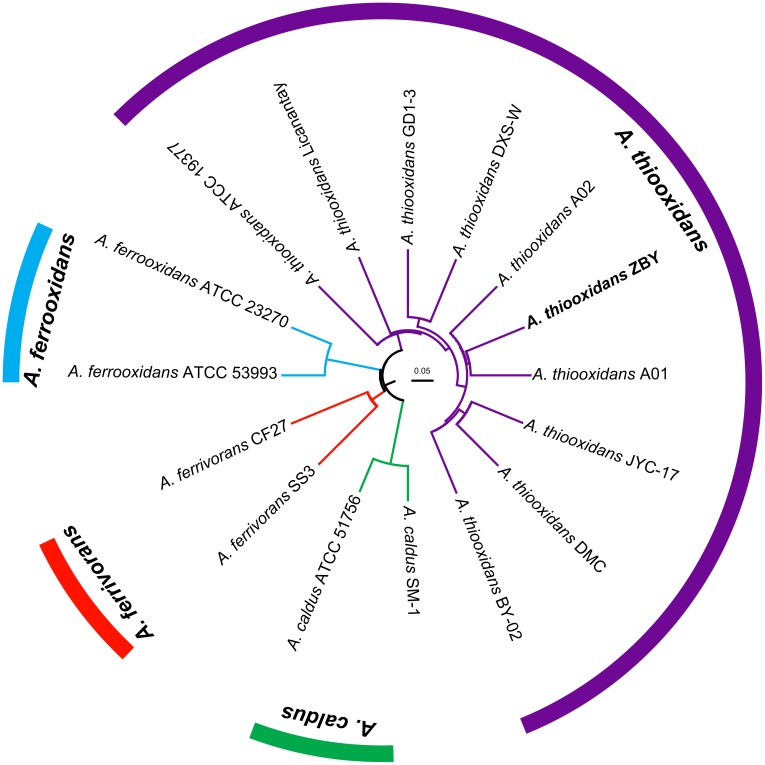
Whole-genome-based phylogeny depicting the phylogenetic relationships amongst bacterial strains of *Acidithiobacillus* spp. CVTree3 with a composition vector approach was applied and *Leptospirillum ferrooxidans* C2-3 was used as an out-group.

Strategy for phylogenetic construction was employed to assess the relationships between newly sequenced strain originally isolated from Zambian copper mine and other known *Acidithiobacillus* spp. deposited in public database. A dendrogram based on 16S rRNA gene sequences showed that the novel strain was clustered on a distinct branch within the identified *A. thiooxidans* species, but was clearly distinguished from other *Acidithiobacillus* isolates including *A. ferrooxidans*, *A. ferrivorans*, and *A. caldus* (unpublished data). Accordingly, we preliminary inferred that strain ZBY in this study was likely to be assigned to *A. thiooxidans* species. Since species identification at the 16S rRNA gene level has its limitation, a genome-based phylogeny was further conducted implementing the CVTree3 web server (**Figure 1**). In addition, ANIb and ANIm, and TETRA were further determined to infer the phylogenetic relationships between strain ZBY and other *Acidithiobacillus* strains (Supplementary Figure S2). High values of ANIb (≥97.27%), ANIm (≥97.88%), and TETRA (≥0.995) between pairs indicated that strain ZBY was very closely related to all recognized *A. thiooxidans* strains. The results combined with whole-genome-based phylogeny strongly supported the prior notion of 16S rRNA sequence-based phylogenetic tree. Taken together, we inferred that strain ZBY was phylogenetically affiliated with *A. thiooxidans* species.

### Pan-Genome Analysis of *A. thiooxidans* Strains

**FIGURE 2:**
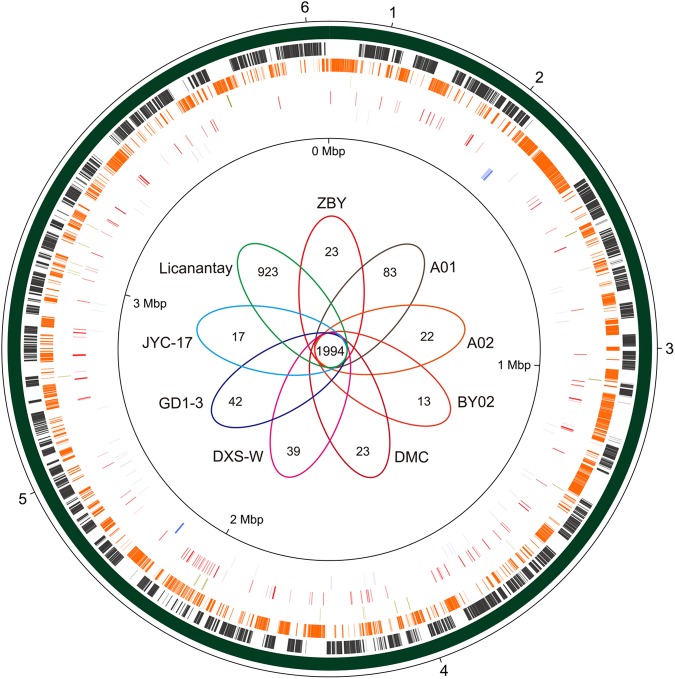
Circular representation of *A. thiooxidans* pan-genome. BLASTN-based whole genome comparisons of *A. thiooxidans* strains were performed and visualized using Circos. Here the newly sequenced strain ZBY was used as a reference. The predicted core genome based on nine strains of *A. thiooxidans* was shown in the second ring. Moving inward, dispensable genome and ZBY-specific genes were indicated in the following two rings (more details for the strategy of strain selection are shown in section “Materials and Methods”). In addition, transposases and tRNA were shown on the fifth and sixth circles. Venn diagram depicting the orthologous and strain-specific CDSs of *A. thiooxidans* strains was shown in the figure center. Several genomic regions of interest were numbered with a pan-genome locus (PL) of 1–6, including *nirBD*/*nasA* (1), carboxysome-associated gene cluster (2), hydrogenase-like gene cluster 2 (3), *narGHJI* (4), hydrogenase-like gene cluster 1 (5), and *coxLMS* (6). PL 1–6 were linked to **Figure 5** and Supplementary Figures S4A–D, S5, respectively.

BLASTN-based whole genome comparisons were performed to identify the core and dispensable genomes among these *A. thiooxidans* strains. Circular plot depicting genome structure was visualized using the software Circos (**Figure 2**). Pan-genome analysis based on nine *A. thiooxidans* strains was conducted, showing that there were a total of 5,999 genes, in which 1,994 were identified to be shared genes (core genome; **Figure 2**). Further inspection showed that the percentages of shared genes accounting for each genome vastly varied, ranging from 52.0% (DXS-W) to 54.8% (A02). The non-core genes (1,719) within *A. thiooxidans* ZBY were assigned to dispensable genome, which contains genes shared by a subset of *A. thiooxidans* genomes and strain-specific genes. Interestingly, relatively few strain-specific genes (23) unique in strain ZBY were predicted compared to that in strain Licanantay (923). COG classification showed that the most abundant strain-specific genes in these strains were assigned to COG categories [L], while was in line with previous studies (Travisany et al., 2014; Zhang et al., 2016a).

### Mathematical Extrapolation for *A. thiooxidans* Pan-Genome

As stated by Tettelin et al. (2005), one or two bacterial genomes per species might provide insufficient information to understand its genetic variability. Vernikos et al. (2015) also recommended that at least five genomes were necessary for mathematical extrapolation. Accordingly, strains A01 (Yin et al., 2014b), Licanantay (Travisany et al., 2014), A02, BY-02, DMC, DXS-W, GD1-3, JYC-17 (Zhang et al., 2016a), and the novel strain ZBY, which was sequenced as part of our present study, were chosen for comparison survey. Genome sizes of these nine strains varied from 3.72 (A02) to 3.95 Mbp (DXS-W; Supplementary Table S2). The completeness values of ≥98.72% supported the mathematical modeling of *A. thiooxidans* pan-genome.

**FIGURE 3:**
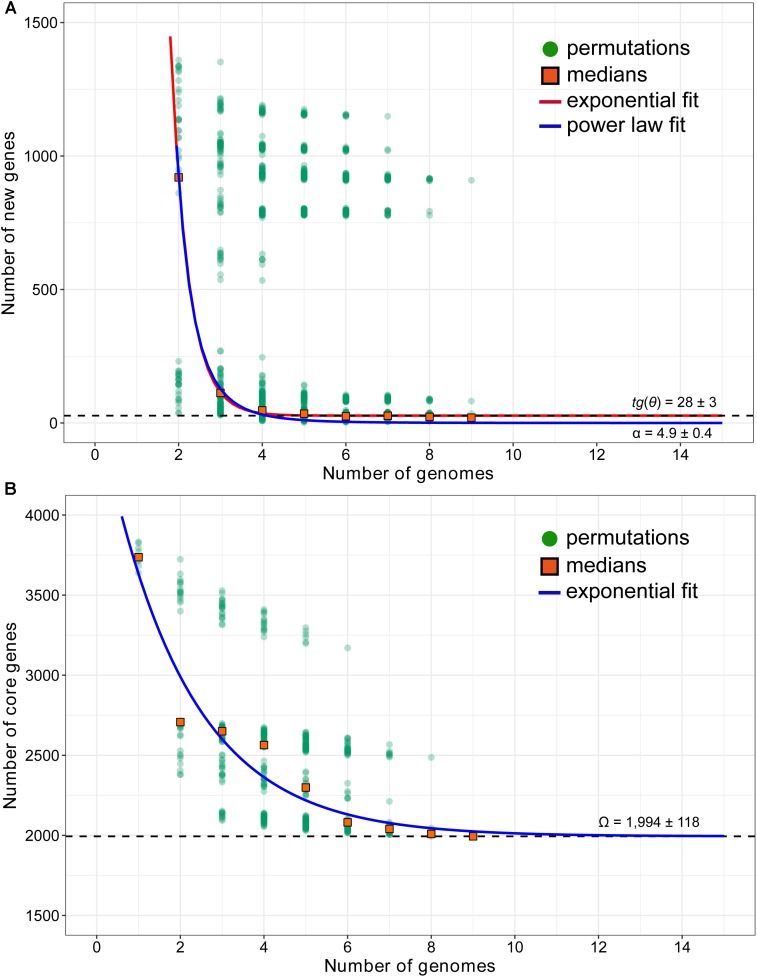
Mathematical extrapolation estimating the new genes **(A)** and core genome **(B)** of *A. thiooxidans* species, based on these sequenced genomes of bacterial strains (except for ATCC 19377). In the new genes extrapolation, new genes were counted by the growth of the gene pool of (*n* – 1) strains when they were added. For each *n*, there are *N*_2_ = *n*⋅*N*_1_ observations. Orange squares are the medians of such values presented in *n* strains. The curve in main window represents the least-squares fit of *F_s_* = 𝜀*_s_*exp(–*n*/τ*_s_*) + *tg*(𝜃). The optimal fitting was output with adjust R-square = 0.9996 for 𝜀*_s_* = 93,320 ± 16,520, τ*_s_* = 0.43 ± 0.04, and *tg*(𝜃) = 28 ± 3. The dashed line displays the extrapolated growth rate of pan-genome *tg*(𝜃). The curve in child window represents the growth curve of pan-genome as the function *P*(*n*). In addition, the blue curve is least-squares fit of the power law *P_s_* = κ*n*^-α^ to medians. A threshold parameter (α) is used to distinguish whether the *A. thiooxidans* pan-genome is open (α ≤ 1) or closed (α > 1); as for core gnome extrapolation, the number of core genes shared by *n* of strains was plotted. For each *n*, there are *N*_1_ = S!/[(*n* – 1)!⋅(S – *n*)!] observations, where *S* is the numbers of strains. Orange squares are the medians of such values presented in *n* strains. The blue curve represents the fit least-squares of *F_c_* = 𝜀*_c_*exp(–*n*/τ*_c_*) + Ω. The optimal fitting was output with adjust R-square = 0.9912 for 𝜀*_c_* = 2,690 ± 421, τ*_c_* = 2.0 ± 0.54, and Ω = 1,994 ± 118. The dashed line displays the extrapolated size of core genomes Ω.

To predict the pan-genome size of *A. thiooxidans* species, the number of specific genes with each new sequenced strain was calculated. Mathematical extrapolation of nine genomic data indicated that an average of 690 novel genes were added when a second genome was sequenced (Supplementary Figure S1). The new genes decayed with new sequenced genome, and quickly converge to a non-zero asymptotic value of 28 (**Figure 3A**). Heaps’ law model revealed that the increase of new genes became harder as sampling proceeded (α = 4.9 ± 0.4, estimate ± SE; Supplementary Table S4), suggesting that *A. thiooxidans* pan-genome was predicted to be closed. In addition, the number of shared genes exponentially decayed with the sequential addition of each new genome (**Figure 3B**). The extrapolated curve indicated that the number of core genes reached a constant number (1,994 ± 118) when the ninth genome was added.

### Functional Classification of the Core and Dispensable Genomes

Functional classification analysis of core and dispensable genomes was carried out by comparison against the extended COG database (Supplementary Figure S3). As expected, relatively high proportion of genes dispersed in the core genome (31.13%) were predicted to be involved in metabolic profiles compared to that in dispensable genome (11.43%). In particular, functional categories [C] (energy production and conversion; 7.17%), [E] (amino acid transport and metabolism; 6.24%), and [G] (carbohydrate transport and metabolism; 4.16%) were abundant in core genome, highlighting the importance of essential genes for basic activities of bacterial strains. In contrast, more genes within dispensable genome were assigned to COG categories [L](replication, recombination, and repair; 8.28%) and [U] (intracellular trafficking, secretion, and vesicular transport; 3.85%) in comparison with that in core genome (4.21% and 1.75%, respectively). In addition, vast genes were predicted to encode hypothetical proteins with unidentified functions, which still need to be further explored.

### Linking Core Genome to the Inferred Metabolic Traits

Identification of predicted metabolic profiles of *A. thiooxidans* genomes was conducted, providing further insights into metabolic traits of these microorganisms. KAAS was applied to investigate the metabolic potentials of all strains (Supplementary Table S5). A large number of core genes were assigned to carbohydrate metabolism (172), amino acid metabolism (142), and energy metabolism (133); this result was consistent with COG classification. In the following sections, we focus on central metabolism of *A. thiooxidans* strains.

#### Central Carbon Metabolism

**FIGURE 4:**
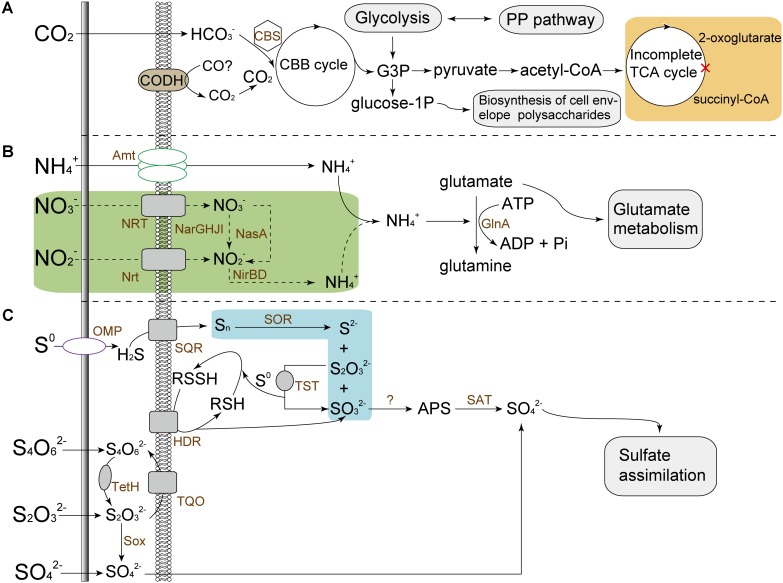
Predicted models for central metabolisms of *A. thiooxidans* strains, including carbon assimilation **(A)**, nitrogen metabolism **(B)**, and sulfur oxidation **(C)**. In the incomplete TCA cycle (citrate cycle), 2-oxoglutarate dehydrogenase that catalyzes the transformation of 2-oxoglutarate to succinyl-CoA was predicted to be absent (orange area), which were also previously reported in some other studies (Zhang et al., 2016a,e). The nitrate reductase (NarGHJI) and nitrite reductase (NirBD) pertaining to dissimilatory nitrate reduction, and putative assimilatory nitrate reductase catalytic subunit (NasA) associated to assimilatory nitrate reduction were predicted to be present in *A. thiooxidans* species except for ATCC 19377 (green area). For strain ATCC 19377, ammonium was required as an alternative nitrogen source. Unlike other *A. thiooxidans* strains, a key enzyme sulfur oxygenase reductase (SOR) involved in sulfur oxidation was absent in draft genome of strain ATCC 19377 probably due to the low sequencing depth (blue area). CBS, carboxysome; PP pathway, pentose phosphate pathway; CODH, carbon-monoxide dehydrogenase; CBB cycle, Calvin–Benson–Bassham cycle; G3P, glyceraldehyde-3-phosphate; Amt, ammonia transporter; Nrt, nitrate/nitrite transporter; NRT, nitrate transporter; NarGHJI, nitrate reductase; NirBD, nitrite reductase; NasA, assimilatory nitrate reductase catalytic subunit; GlnA, glutamine synthetase; OMP, outer membrane protein; SQR, sulfide quinone oxidoreductase; TQO, thiosulfate:quinone oxidoreductase; TetH, tetrathionate hydrolase; Sox, sulfur oxidizing protein; TST, thiosulfate sulfurtransferase; HDR, heterodisulfide reductase.

The assimilation of carbon source derived from external environments is essential for organisms. Similar to many other autotrophic acidophiles (Valdés et al., 2008; Ullrich et al., 2016; Zhang et al., 2016a,b,e), *A. thiooxidans* isolates have the ability to fix atmospheric CO_2_ via the classical Calvin–Benson–Bassham (CBB) cycle (**Figure 4**). For carbon fixation, ribulose-1,5-biphosphate carboxylase/oxygenase (Rubisco) directing the regeneration of ribulose-1,5-bisphosphate was commonly recognized as an crucial enzyme in CBB cycle(Zhang et al., 2016c,e). All *A. thiooxidans* strains were predicted to harbor a carboxysome-associated gene cluster, which potentially encodes Type I Rubisco large and small subunits, carboxysome shell carbonic anhydrase, several copies of carboxysome shell proteins and carbon dioxide-concentrating proteins (Supplementary Figure S4A).

Glyceraldehyde-3-phosphate (G3P), one of important metabolic intermediates in the process of carbon fixation, was predicted to be involved in the formation of certain precursors for the biosynthesis of amino acids, fatty acids, and polysaccharides via central metabolism. *A. thiooxidans* genomes were predicted to harbor genes involved in amino sugar and nucleotide sugar metabolism (ko00520; Supplementary Table S5). Of note, glucose-6-phosphate and glucose-1-phosphate converted by G3P might be involved in the conversion of precursors for the biosynthesis of cell envelope polysaccharides (Supplementary Table S6), as previously described by Ullrich et al. (2016). Additionally, a three-gene cluster (*coxLMS*) encoding putative carbon-monoxide dehydrogenase (EC: 1.2.7.4) were identified in *A. thiooxidans* genomes (Supplementary Figure S4B).

#### Nitrogen Assimilation

Unlike *A. ferrooxidans* (Valdés et al., 2008), no similar components of nitrogenase complex were identified in *A. thiooxidans* isolates. Comparisons of the metabolic profiles of all *A. thiooxidans* strains revealed that these microorganisms except for ATCC 19377 harbor genes for dissimilatory nitrate reduction, observed as *nirBD* and *narGHJI* (Supplementary Figures S4C, D, respectively). As for assimilatory nitrate reduction, gene *nasA* encoding putative nitrate reductase (NADH) catalytic subunit was found in all *A. thiooxidans* strains except for ATCC 19377. Besides, the subunit NasB that transfers electrons derived from NADH to nitrate (Lin and Stewart, 1997) was absent in *A. thiooxidans* genomes, thereby making the electron donor unclear. In addition, genes coding for nitrate and/or nitrite transporter were absent in *A. thiooxidans* ATCC 19377.

All *A. thiooxidans* strains were predicted to harbor the ammonium transporters to take up the external ammonia, and to assimilate the latter into central metabolic pathways by a series of enzymes, such as glutamine synthetase and glutamate synthase (**Figure 4**).

#### Sulfur Oxidation and Electron Transfer

According to COG class assignment, numerous genes pertaining to the core genome of *A. thiooxidans* species were predicted to be related to energy production and conversion (COG category [C]; Supplementary Figure S3). Oxidation of various sulfur species (e.g., tetrathionate and elemental sulfur) was a well-studied characteristic of *A. thiooxidans* strains (Yin et al., 2014a; Zhang et al., 2016a). In this study, the core genome was replete with enzymes involved in sulfur oxidation, such as sulfide quinone oxidoreductase, tetrathionate hydrolase, thiosulfate:quinone oxidoreductase, sulfur oxidizing protein system, and sulfur oxygenase reductase (**Figure 4**). However, the *sor* gene was absent in *A. thiooxidans* ATCC 19377, possibly due to the low-coverage genome sequencing (Zhang et al., 2016e). Also, a number of genes within core genome of *A. thiooxidans* species were predicted to be involved in electron transport chain, including *cydAB*, *cyoABCD*, and *nuoABCDEFGHIJKLMN* (Supplementary Table S7).

#### Hydrogenase-Like in *A. thiooxidans* Strains?

Numerous genes involved in energy metabolism were predicted to be distributed in the core genome of *A. thiooxidans*. Notably, several hydrogenase-like genes were observed in all sequenced *A. thiooxidans* genomes (sections 3 and 5 in **Figure 2**). In light of previous report showing that *A. thiooxidans* strains could not grow chemolithotrophically on hydrogen (Hedrich and Johnson, 2013b), the genotypic traits appear to be inconsistent with their phenotypes. To address this issue, the following sections focus on two specific genomic regions containing hydrogenase-like genes.

**FIGURE 5:**
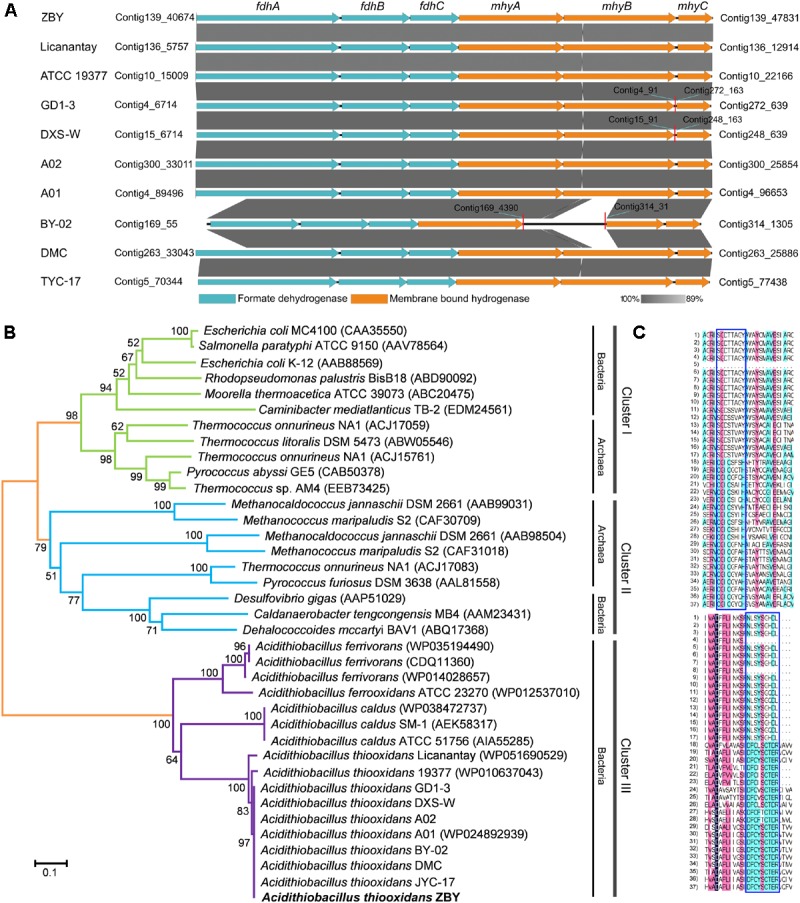
Identification of the putative formate hydrogenlyase in *A. thiooxidans* strains. **(A)** Visualization for the putative *fdh*–*mhy* gene cluster of *A. thiooxidans* strains using the software EasyFig. **(B)** Phylogenetic tree showing the relationships between *Acidithiobacillus* spp. and other organisms having the group 4 [NiFe]-hydrogenases. The tree was based on the large subunits of putative hydrogenases from *Acidithiobacillus* spp. plus various large subunits of recognized hydrogenases from different organisms including *Bacteria* and *Archaea*, and was generated and visualized using the MEGA. The neighbor-joining tree was constructed with a bootstrap analysis implementing 1,000 replicates. **(C)** Sequence representations of conserved motifs (in the blue boxes) from various organisms.

Hydrogenases (H_2_ases) directing the interconversion of molecular hydrogen (Vignais et al., 2001; Jenney and Adams, 2008; Lim et al., 2010) are widely distributed in all domains of life. They are believed to play a central role in the biological production as well as hydrogen utilization. According to the classification of H_2_ases (Vignais et al., 2001), three H_2_ases in all *A. thiooxidans* strains seem to be assigned to the putative membrane-bound H_2_ evolving [NiFe]-H_2_ases (group 4). A six-gene cluster was predicted to encode the H_2_ases complex, which consists of two H_2_ases, two NADH-ubiquinone oxidoreductases, a NADH dehydrogenase, and a formate dehydrogenase. Further inspection showed that these genes were organized into a bipartite *fdh*–*mhy* cluster, including three *fdh* genes coding for formate dehydrogenase and three *mhy* genes coding for membrane-bound H_2_ases (**Figure 5A**). To evaluate the phylogenetic relationship of H_2_ases, the large subunits encoded by *mhyB* genes from various species were selected for further analysis. A phylogeny demonstrated that these targeted H_2_ases were obviously separated into three clusters (**Figure 5B**), in which clusters I and II were assigned to groups 4b and 4a, respectively, as previously described by Lim et al. (2010). Notably, H_2_ases in *Acidithiobacillus* spp. (cluster III) were significantly distinct from those in other species, probably indicating a novel enzyme complex. In support of this designation, DNAMAN program was employed to perform the multiple sequence alignment. As depicted in **Figure 5C**, two conserved motifs (SGD[TS][TS][AV][GA]Y and NLSYSGHDL) were identified in all *Acidithiobacillus* spp.

Another gene cluster potentially encoding formate dehydrogenase, H_2_ase, NADH dehydrogenase, two copies of NADH-ubiquinone oxidoreductase, and H_2_ase was also identified in *A. thiooxidan*s genomes. These six genes were assigned as new labels (*orf1*–*orf6*), and compared and visualized using the software EasyFig (Supplementary Figure S5A). To further analyze the possible function of these candidate genes, the corresponding amino acid sequences were extracted for the identification of conserved domains. The results confused us as some conserved domains were involved in the [NiFe]-H_2_ases, whereas others were associated with the NADH-ubiquinone oxidoreductase (Supplementary Figure S5B). In order to identify their putative functional properties, amino acid sequences from *A. thiooxidans* ZBY were aligned to other complex, including H_2_ase Group III and IV and NADH-ubiquinone oxidoreductase. Results showed that *orf4* and *orf5* was potentially annotated as the large and small subunits of both Group III and IV [NiFe]-H_2_ases, respectively (Supplementary Figure S5C). According to the conserved regions of [NiFe]-H_2_ase groups (Vignais et al., 2001), however, no L1 and L2 signature was identified in these large subunits (unpublished).

## Discussion

In our mathematical model, a threshold parameter (α > 1) indicated that the pan-genome of *A. thiooxidans* species was closed (**Figure 3** and Supplementary Table S4). Some microbial species with a closed pan-genome, such as *Bacillus anthracis* and *Mycobacterium tuberculosis*, inhabit the isolated environments with limited access to the existing global microbial gene pool, suggesting the genome stability and relatively conserved genome (Medini et al., 2005). Aside from core genome, the remaining dispensable genes contribute to species’ genome diversity. In other microorganisms, studies have discussed issues related to the correlation between the dispensable genome and genetic variation (Lindsay et al., 2012; Graña-Miraglia et al., 2017). In comparison with microbial species harboring an open pan-genome, such as *Leptospirillum ferriphilum* (Zhang et al., 2018), the size of *A. thiooxidans* pan-genome would reach a plateau as a certain number of genomes were sampled. For species with a closed pan-genome, additional sequenced genomes would not expand the species’ pan-genome, showing a relatively limited gene content diversity (Tettelin et al., 2008).

Clusters of Orthologous Groups classification revealed that relatively more core genes within *A. thiooxidans* were assigned to COG categories [C], [E], and [M] compared to dispensable genes (Supplementary Figure S3). The COG classes [C] and [E] are involved in metabolisms of energy and amino acids, and COG class [M] is associated with the biogenesis of cell wall/membrane/envelope. Accordingly, we inferred that efficient uptake of energy and nutrients from acidic environments was essential for the autotrophic lifestyle of acidophile *A. thiooxidans*, and specialized cellular structures might provide a selective advantage to adapt to the harsh econiches. Further inspection showed that genes associated with COG class [L] in dispensable genome were more than that in core genome (Supplementary Figure S3). We therefore interpret this as an indication that the large proportion of genes involved in COG category [L] might endow *A. thiooxidans* with adaptive strategies to respond to the adverse environments, as high concentrations of toxic substances such as heavy metal elements in acidic bioleaching operations tend to cause DNA injury (Silver and Phung, 1996). Once cell damage occurs, the rescue system may play a critical role in DNA repair. The findings presented here were in line with the previous studies (Travisany et al., 2014; Zhang et al., 2016a), highlighting an adaptive advantage in individual biomining environments. Taken together, our results showed that dispensable genome might provide microorganisms with functions that were essential to niche adaptation (Tettelin et al., 2008).

Unlike other acidophilic bacteria such as *Sulfobacillus* spp. that utilize both inorganic and organic forms of carbon (Guo et al., 2014; Justice et al., 2014; Zhang et al., 2017) and *Leptospirillum* spp. that assimilate carbon via the reductive tricarboxylic acid cycle (Levicán et al., 2008; Zhang et al., 2016c), *A. thiooxidans* was known to be autotrophic carbon fixer that assimilates carbon via classical CBB cycle. In a gene cluster related to carboxysome, one gene was predicted to encode a novel carbonic anhydrase (CA; 𝜀-class CA, Zhang et al., 2016e). The carboxysome-associated carbonic anhydrase elevates the concentration of carbon dioxide near the Rubisco by the conversion of accumulated cytosolic bicarbonate into CO_2_ (Price et al., 2004), thereby suggesting a more efficient CO_2_ fixation.

With respect to nitrogen assimilation, in general, microorganisms-mediated inorganic nitrogen metabolisms are commonly involved in the uptake of atmospheric nitrogen, ammonium, nitrite, and nitrate (Arrigo, 2005). The closely related *A. ferrooxidans* was reported to harbor the diazotrophic lifestyle (Valdés et al., 2008; Zhang et al., 2016c). By contrast, the dissimilatory and assimilatory nitrate reduction in *A. thiooxidans* species (except for ATCC 19377) could be an alternative pathway to acquire nitrogen source. Additionally, genes associated to nitrate and/or nitrite transporter were absent in strain ATCC 19377. Although genes/gene clusters pertaining to dissimilatory and assimilatory nitrate reduction were identified in a number of *A. thiooxidans* strains, we could not determine whether these genes/gene clusters were introduced by horizontal gene transfer (HGT), since no mobile genetic element, such as transposases, integrase, and tRNA gene, was found in the neighborhood of these observed genomic regions. In other words, we inferred that the absence of the corresponding genes/gene clusters in strain ATCC 19377 was more likely caused by gene loss rather than HGT. In other organisms, many studies have focused on genome reduction in the condition of increased biological fitness and efficient utilization of limited nutrients especially in oligotrophic ecosystems (Boon et al., 2014; Giovannoni et al., 2014; Martínez-Cano et al., 2015; Zhang et al., 2017). Given that the concentrations of nitrogen resources (such as ammonium, nitrate, and nitrite) are very limited in many acidic eco-environments (Parro et al., 2007; Chen et al., 2015), the genome reduction might provide a selective advantage that drives the abandonment of expensive genes, supporting the effective acquisition of limited nutrients. Therefore, the abandonment of these dispensable genes might increase the cellular economization, reference to a streamlining hypothesis (Mira et al., 2001; Dufresne et al., 2005; D’Souza et al., 2014). In addition, the results revealed that not all *A. thiooxidans* strains in the microbial communities should reduce their genome sizes as it is of importance to maintain a functional diversity (Giovannoni et al., 2014). Taken together, our findings supported that similar to gene gain such as HGT, gene loss was the equally important driving force that contributes to the microbial genome evolution. Further analysis of potential DNA donor-recipient is an interesting prospect to investigate the genome evolution by accessing the microbial gene pool of the whole community.

In general, *A. thiooxidans* species was known as the sulfur oxidizer, which has a sulfur-dependent chemolithoautotrophic lifestyle. All of the strains utilize energy and electrons derived from aerobic oxidation of various sulfur species for assimilation of inorganic carbon and other anabolic processes (Yin et al., 2014a; Zhang et al., 2016a,c). Electrons from elemental sulfur and various sulfur compounds are transferred via the quinol pool (i) either to *bd*-type or *bo_3_*-type terminal oxidases, (ii) or to NADH-ubiquinone oxidoreductase (complex I; Yin et al., 2014a). Unlike nitrogen metabolism, in which some genes have undergone the gene loss, genes pertaining to sulfur oxidation were predicted to be dispersed in the core genome of *A. thiooxidans* species, suggesting that these conserved genes might evolve from a common ancestor and were more stable to be responsible for the sulfur oxidation of *A. thiooxidans* species.

The closely related *A. ferrooxidans* (formerly *Thiobacillus ferrooxidans*) not only utilizes the ferrous iron as the electron donor, but also gains energy generated from the oxidation of RISC, hydrogen, and formate to support its growth (Drobner et al., 1990; Pronk et al., 1991; Valdés et al., 2008). Hedrich and Johnson (2013b) further revealed that three species of *Acidithiobacillus* (*A. ferrooxidans*, *A. caldus*, and *A. ferridurans*) that oxidize ferrous iron or sulfur as electron donors could also grow on hydrogen. In their study, however, efforts to reveal the hydrogen utilization of two *A. thiooxidans* strains have been failed, thereby making it questionable whether this species could or could not metabolize hydrogen. Considering the inconsistency between experimental evidence and genomic analyses, we thus inferred that the presence of these hydrogenase-like in *A. thiooxidans* strains might not necessarily imply that the corresponding proteins could perform the predicted function as they might play other roles in bacterial lifestyle. In the following sections, further analyses were attempted to explain this inconsistency.

In the hyperthermophilic archaeon *Thermococcus litoralis*, an eight-gene cluster (*fdhAB*–*mhyCDEFGH*) was reported to encode the formate hydrogenlyase (formate dehydrogenase-coupled H_2_ase, FDH–MHY) complex, which supports microbial non-syntrophic growth on formate and metabolizes the hydrogen (Takács et al., 2008). The FDH–MHY complex consists of a formate dehydrogenase and a membrane-bound [NiFe]-H_2_ase which is considered as the member of H_2_-evolving, energy-conserving, membrane-associated H_2_ases. In addition, *in silico* genomic analysis of *Thermococcus onnurineus* showed the presence of a tripartite *fdh*–*mfh*–*mnh* cluster encoding for formate dehydrogenase, multimeric membrane-bound H_2_ases, and cation/proton antiporter, which was classified as the subgroup 4b of [NiFe]-H_2_ase (Kim et al., 2010; Lim et al., 2010). Further analysis showed that a pair of highly conserved hydrogen and nickel-binding motifs (C[GS][ILV]C[AGNS]xxH and [DE][PL]Cx[AGST]Cx[DE][RL], “x” represents any amino acid) were identified in the large subunits of H_2_ases (Wu and Mandrand, 1993; Lim et al., 2010). Although two conserved domains were predicted in all putative group 4 H_2_ases of *A. thiooxidans* strains used in our study, they were quite distinct from the conserved motifs in catalytic large subunits of other known Group 4 [NiFe]-H_2_ases, as some variant residues were identified in the former. The similar results were also reported in several other acidophiles (Yelton et al., 2013; Justice et al., 2014). In their studies, the putative group 4 H_2_ases were thought to perform some other function due to the absence of the binding site motifs that were normally found in [NiFe]-H_2_ases and were considered to be necessary for H_2_ formation (Wu and Mandrand, 1993; Lim et al., 2010).

Previous studies revealed the identical order and organization, and sequence similarities of certain essential subunits of [NiFe]-H_2_ases with subunits of energy-conserving NADH-ubiquinone oxidoreductase (also called respiratory complex I, Albracht et al., 1997; Hedderich, 2004). However, the reaction catalyzed by multi-subunit membrane-bound [NiFe]-H_2_ases was significantly different from the reaction catalyzed by complex I (EC: 1.6.5.3, Hedderich, 2004). The former is responsible for the interconversion of hydrogen, whereas the latter catalyzes the electron transfer from NADH to ubiquinone (Albracht et al., 1997; Friedrich and Scheide, 2000). In light of the six subunits (NuoB, C, D, H, I, and L) common to complex I and membrane-bound [NiFe]-H_2_ases, Friedrich and Scheide (2000) proposed a hypothesis that the complex enzyme consisting of the peripheral subunits (NuoB, C, D, and I) and the membraneous subunits (Nuo H and L) might be the common ancestor of complex I and multi-subunit membrane-bound H_2_ases. On the basis of the aforementioned evolutionary relationship of complex I and membrane-bound [NiFe]-H_2_ases, we inferred that the enzyme complexes in *A. thiooxidans* strains might evolve from an ancestral enzyme of complex I and recognized [NiFe]-H_2_ases, but have some other unclear functional roles.

## Conclusion

Pan-genome analysis presented here expands our current understanding of genetic characteristics of *A. thiooxidans* isolates. Mathematical extrapolation revealed a closed pan-genome of *A. thiooxidans* species. Comparisons of gene repertoire, especially metabolic profile, of *A. thiooxidans* strains provide new insights into their autotrophic lifestyle, which strengthen our recognition that *A. thiooxidans* is an obligately chemolithoautotrophic acidophile capable of assimilating the atmospheric CO_2_ and inorganic forms of nitrogen, acquiring energy from aerobic oxidation of various sulfur species.

## Author Contributions

XZ drafted and wrote the manuscript, and performed the pan-genome analysis. ZL carried out the mathematical modeling. GW, FY, and XL participated in the discussion.

## Conflict of Interest Statement

The authors declare that the research was conducted in the absence of any commercial or financial relationships that could be construed as a potential conflict of interest.
