# Supplementary material for: In Silico Genome-Wide Analysis Reveals the Potential Links Between Core Genome of Acidithiobacillus thiooxidans and Its Autotrophic Lifestyle
Source: Front Microbiol. 2018 Jun 8;9:1255. doi: 10.3389/fmicb.2018.01255 (PMC6002666; doi:10.3389/fmicb.2018.01255)
Supplement: Supplementary file 1 [file Data_Sheet_1.pdf]

Supplemental Material for

In silico genome-wide analysis reveals the potential links between core genome of *Acidithiobacillus thiooxidans* and its autotrophic lifestyle

Xian Zhang,[1] Zhenghua Liu,[2] Guanyun Wei,[3] Fei Yang,[1] Xueduan Liu,[2,4*]*

[*]Corresponding author: Fei Yang and Xueduan Liu

[1]*Department of Occupational and Environmental Health, Xiangya School of Public Health, Central South University, Changsha, China;*

[2]*School of Minerals Processing and Bioengineering, Central South University, Changsha, China;*

[3]*College of Life Science, Nanjing Normal University, Nanjing, China*

[4]*Key Laboratory of Biometallurgy of Ministry of Education, Central South University, Changsha, China*

SUPPLEMENTARY FIGURES

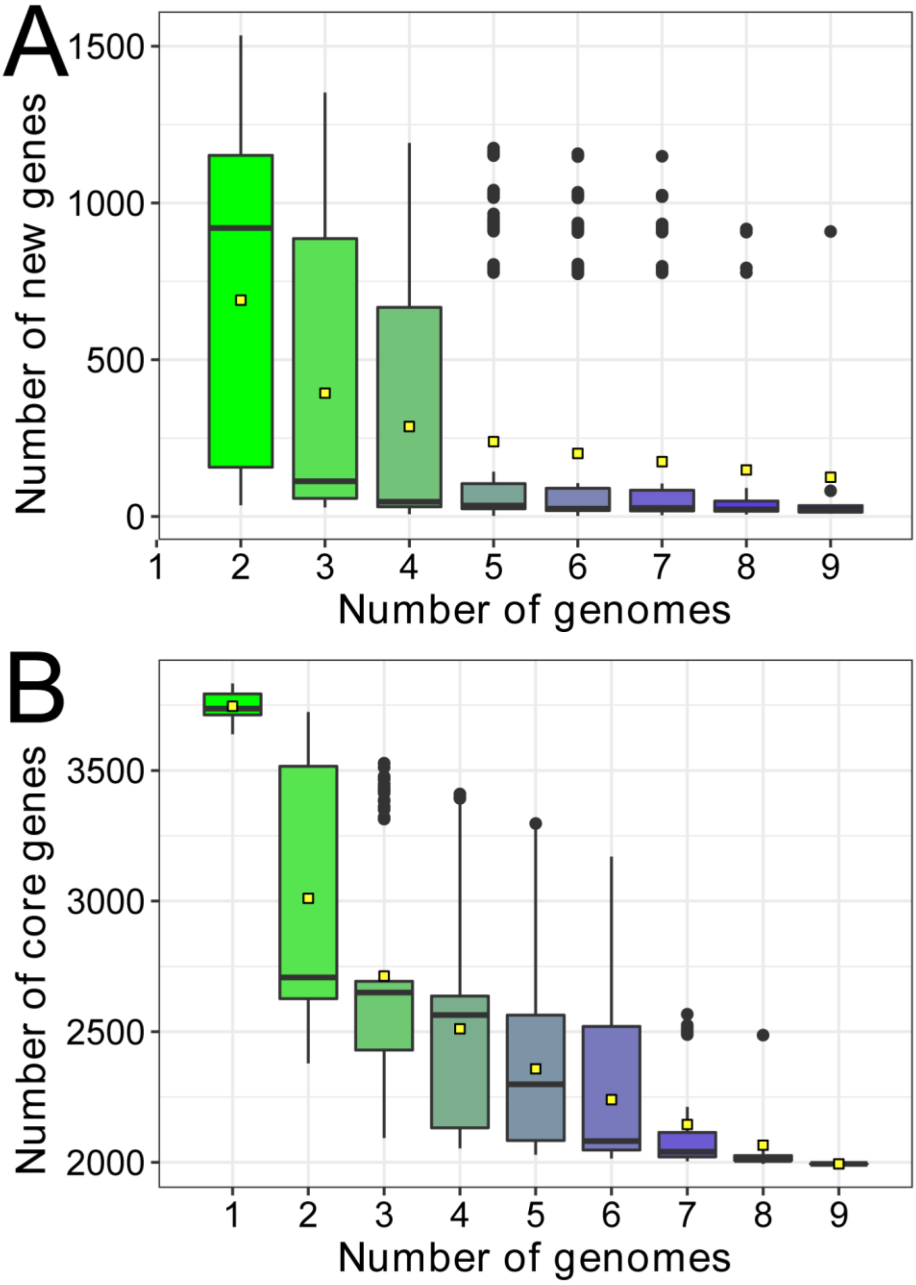

FIGURE S1 Distributions of new genes and core genes' values: averages vs. medians. Dark spots represent the distributions of new genes and core genes' values. For each *N* (the number of sequenced genomes), the box-plots indicating the medians (black lines) and the average (yellow rectangle) were shown.

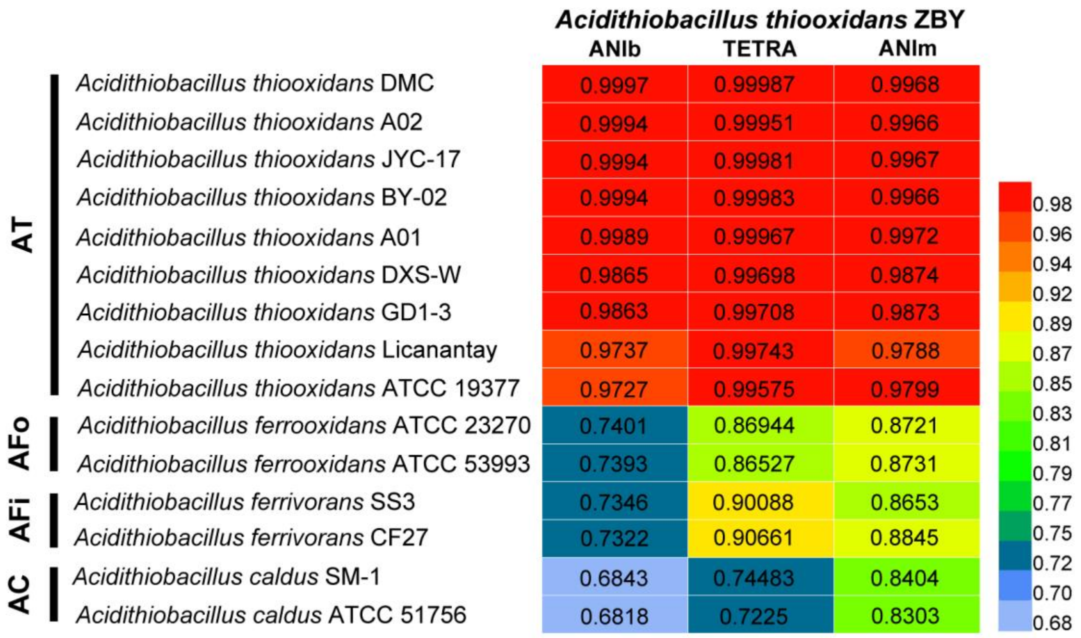

FIGURE S2 Genome-based phylogenetic indicators of *Acidithiobacillus* spp. The average nucleotide identity (ANI) based on BLAST (ANIb) and MUMmer (ANIm), as well as tetranucleotide frequency correlation coefficient (TETRA) were calculated using the software JSpecies v1.2.1 with default parameters. Values above the thresholds of ≥ 95% (ANI) and ≥ 0.99 (TETRA) indicate that strains belong to the same species. Abbreviation: AT, *Acidithiobacillus thiooxidans*; AFo: *Acidithiobacillus ferrooxidans*; AFi: *Acidithiobacillus ferrivorans*; AC: *Acidithiobacillus caldus*.

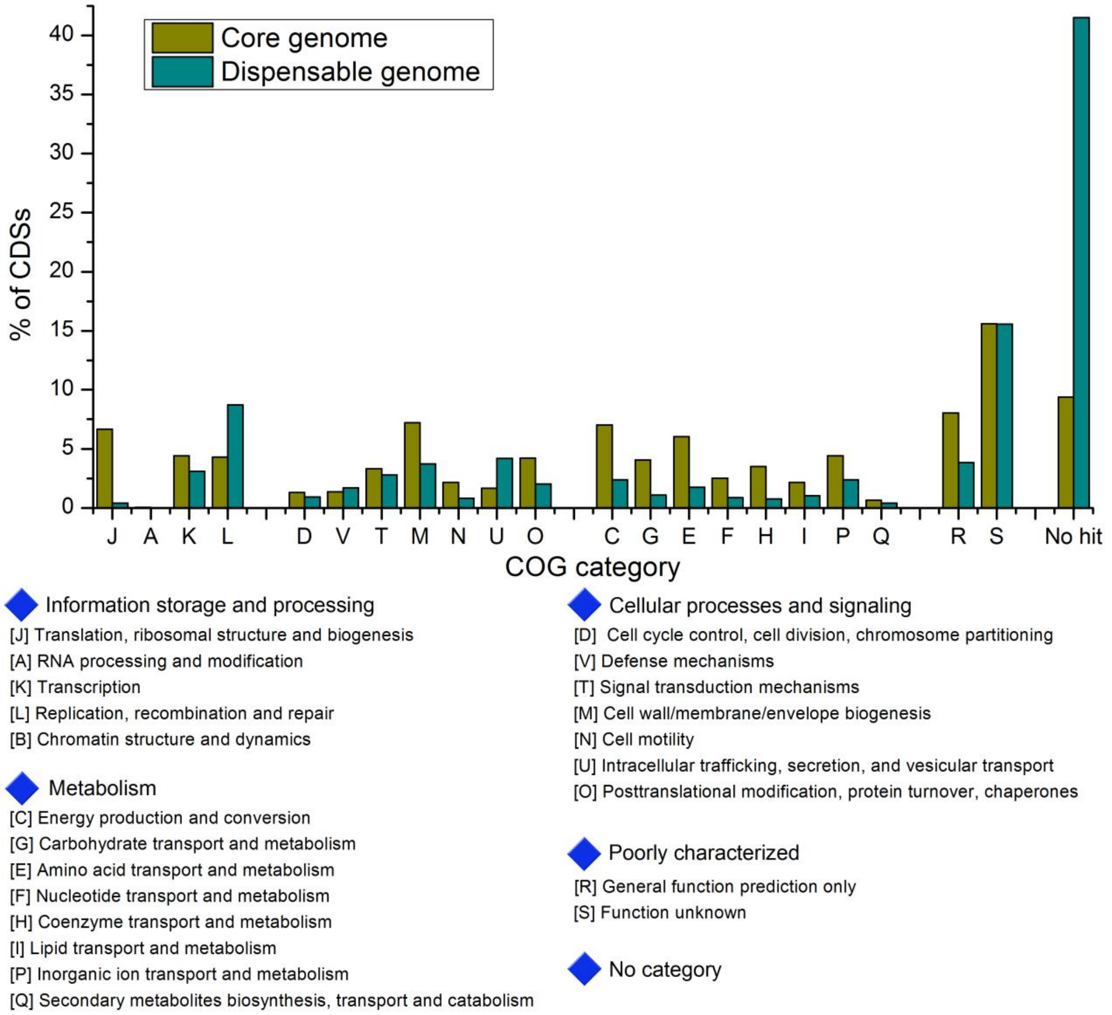

FIGURE S3 Functional assignments of core and dispensable genome via alignment against the extended COG database.

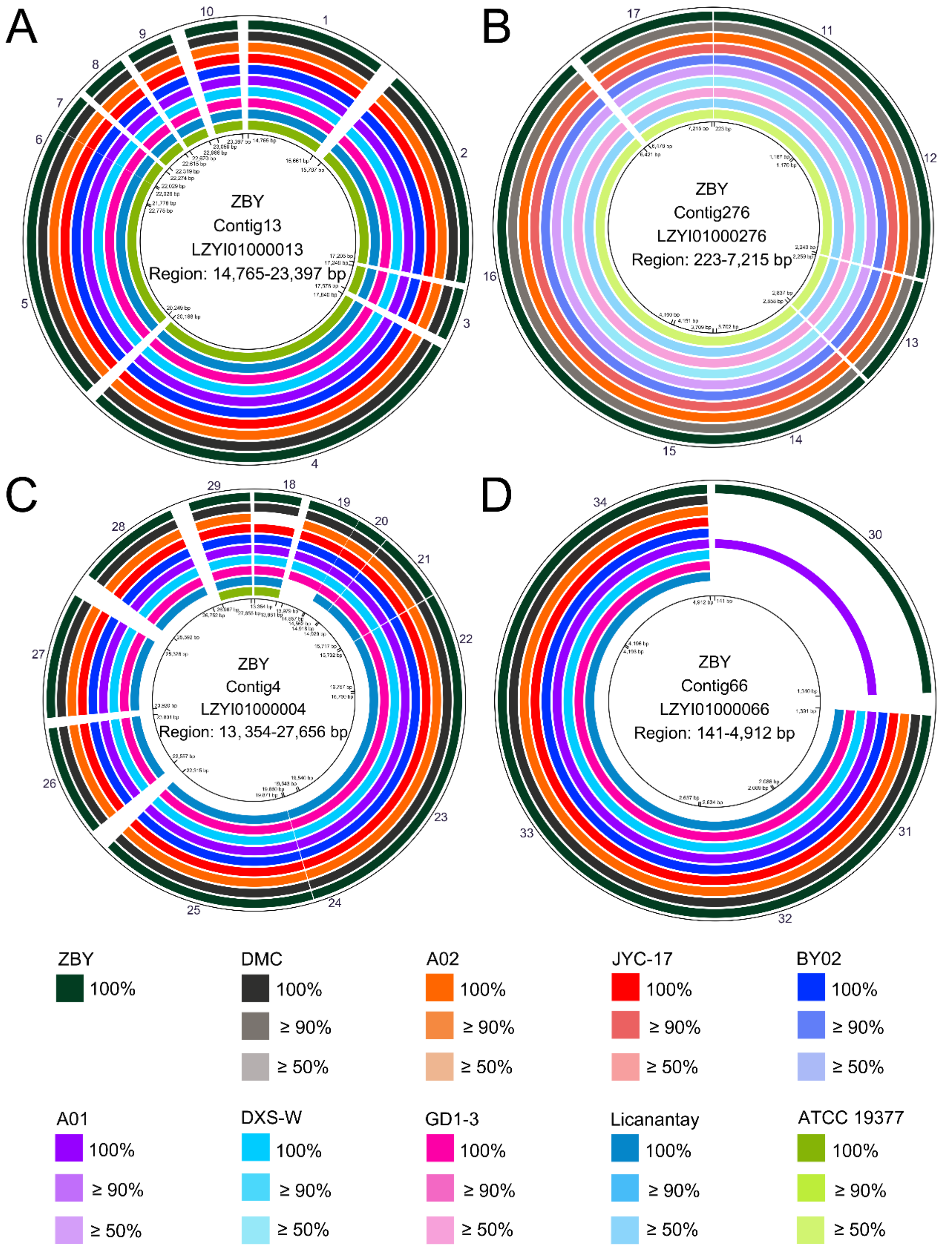

FIGURE S4 Partial genomic map of *A. thiooxidans* strains containing carboxysome-associated gene cluster (A), *coxLMS* (B), *nirBD*/*nasA* (C), and *narGHJI* (D). Genomic regions of *A. thiooxidans* ZBY were used as the reference. Matches to the reference genome were displayed color-coded on the rings two to ten. Color intensity indicates the level of sequence identities between query genome and reference genome (e.g. higher color intensity means the higher sequence identity). Proteins in different genomic regions of interest were numbered with 1 to 34. Abbreviation: 1, RuBisCO operon transcriptional regulator; 2, ribulose-bisphosphate carboxylase large subunit;

3, ribulose bisphosphate carboxylase small subunit; 4, carboxysome shell protein CsoS2; 5, carboxysome shell carbonic anhydrase; 6, carboxysome peptide A; 7, carboxysome peptide B; 8-10, carbon dioxide-concentrating protein CcmK; 11, xanthine dehydrogenase accessory factor; 12, amidohydrolase; 13, ureidoglycolate hydrolase; 14, carbon-monoxide dehydrogenase medium subunit/xanthine dehydrogenase FAD-binding subunit; 15, carbon-monoxide dehydrogenase small subunit/xanthine dehydrogenase, iron-sulfur binding subunit; 16, carbon monoxide dehydrogenase, large subunit/xanthine dehydrogenase, molybdenum-binding subunit; 17, phosphorylase; 18, hypothetical protein; 19, response regulator NasT; 20, cobalamin biosynthesis protein CbiX; 21, uroporphyrin-III methyltransferase; 22, glycosyl transferase, family 3; 23, assimilatory nitrate reductase (NADH) catalytic subunit NasA; 24, assimilatory nitrite reductase [NAD(P)H] small subunit NirD; 25, assimilatory nitrite reductase [NAD(P)H] large subunit NirB; 26, hypothetical protein; 27, major facilitator superfamily MFS_1, nitrate/nitrite transporter; 28, nitrate transporter; 29, transcriptional regulator, Crp/Fnr family; 30, MFS transporter; 31, respiratory nitrate reductase, gamma subunit NarI; 32, respiratory nitrate reductase delta subunit NarJ; 33, respiratory nitrate reductase beta subunit NarH; 34, respiratory nitrate reductase alpha subunit NarG.

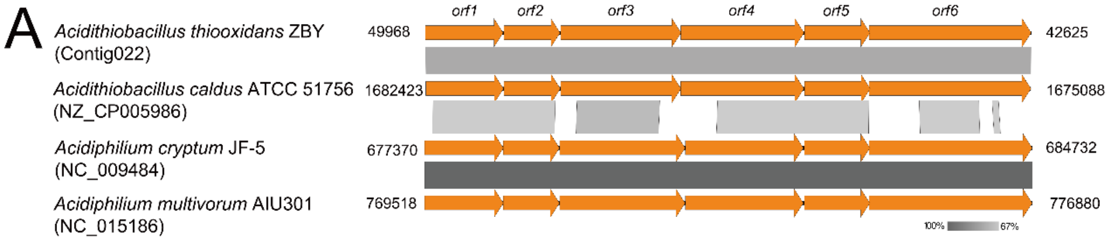

B

Conserved Protein Domain Family		Putative six-gene cluster					
		orf1	*orf2*	*orf3*	*orf4*	*orf5*	*orf6*
Accession	Description	313 aa	226 aa	486 aa	497 aa	263 aa	654 aa
cl00469	NADH dehydrogenase	✓					
cl17899	Hydrogenase-4 membrane subunit HyfE		✓				
PRK06458	Hydrogenase 4 subunit F; Validated			✓			
pfam00361	NADH-ubiquinone/plastoquinone			✓			✓
cl19197	Respiratory-chain NADH dehydrogenase				✓		
COG3261	Ni,Fe-hydrogenase III large subunit				✓		
COG0649	NADH-ubiquinone oxidoreductase subunit D				✓		
cl19102	The HCP family of iron-sulfur proteins					✓	
pfam12838	4Fe-4S dicluster domain					✓	
cl17194	NADH-ubiquinone oxidoreductase					✓	
PRK06521	Hydrogenase 4 subunit B; Validated						✓

C

	orf1	*orf2*	*orf3*	*orf4*	*orf5*	*orf6*
[NiFe]-H2ases						
Group III (*hyc* operon)						
HycD	✓					
HycP		✓				
HycQ			✓			
HycE				✓		
HycG					✓	
HycC						✓
Group IV (*hyf* operon)						
HyfC	✓					
HyfE		✓				
HyfF			✓			
HycG				✓		
HyfI					✓	
HyfB						✓
NADH-ubiquinone oxidoreductase						
NuoH	✓					
NuoD				✓		
NuoB					✓	
NuoL						✓

FIGURE S5 Functional assignments of putative six-gene clusters in *A. thiooxidans* strains. (A) Comparison of *A. thiooxidans* ZBY and other microorganisms containing identified or putative gene cluster; (B) Identification of conserved domains of complex enzyme encoded by six-gene cluster; (C) Comparison of amino acid sequences involved in these candidate genes to the identified [NiFe]-hydrogenases and NADH-ubiquinone oxidoreductase.

SUPPLEMENTARY TABLES

TABLE S1 List of the genomes of novel strain ZBY and other recognized *Acidithiobacillus* (*A.*) spp. used for the construction of genome-based phylogeny and the calculation of average nucleotide identity.

Organism	Accession Number	Genome size (Mb)	GC %	Reference
Strain ZBY	LZYI00000000	3.79	53.17	This study
A. thiooxidans				
ATCC 19377	AFOH00000000	3.02	53.20	(Valdes et al., 2011)
A01	AZMO00000000	3.82	53.10	(Yin et al., 2014)
Licanantay	JMEB00000000	3.94	52.80	(Travisany et al., 2014)
GD1-3	LWSC00000000	3.95	52.92	(Zhang et al., 2016a)
DXS-W	LWRY00000000	3.95	52.92	(Zhang et al., 2016a)
A02	LWSA00000000	3.72	53.01	(Zhang et al., 2016a)
BY-02	LWRZ00000000	3.81	53.07	(Zhang et al., 2016a)
DMC	LWSB00000000	3.85	53.06	(Zhang et al., 2016a)
TYC-17	LWSD00000000	3.83	53.06	(Zhang et al., 2016a)
A. ferrooxidans				
ATCC 23270	NC_011761	2.98	58.80	(Valdés et al., 2008)
ATCC 53993	NC_011206	2.89	58.90	Unpublished
A. caldus				
SM-1	NC_015850	3.24	60.94	(You et al., 2011)
ATCC 51756	NZ_CP005986	2.99	61.39	(Valdes et al., 2009)
A. ferrivorans				
SS3	NC_015942	3.21	56.60	(Liljeqvist et al., 2011)
CF27	CCCS000000000	3.43	56.40	(Talla et al., 2014)

TABLE S2 General features of bacterial strains used for comparative survey in this study.

Strain	Accession number	Genome status	Genome size (Mbp)	Number of CDS	Completeness (%)	Reference
ZBY	LZYI00000000	Draft	3.79	3,713	99.34	This study
A01	AZMO00000000	Draft	3.82	3,719	98.72	(Yin et al., 2014)
Licanantay	JMEB00000000	Draft	3.94	3,793	99.34	(Travisany et al., 2014)
DMC	LWSB00000000	Draft	3.85	3,772	99.34	(Zhang et al., 2016)
A02	LWSA00000000	Draft	3.72	3,639	99.34	(Zhang et al., 2016)
GD1-3	LWSC00000000	Draft	3.95	3,824	99.34	(Zhang et al., 2016)
BY-02	LWRZ00000000	Draft	3.81	3,683	99.03	(Zhang et al., 2016)
JYC-17	LWSD00000000	Draft	3.83	3,737	99.34	(Zhang et al., 2016)
DXS-W	LWRY00000000	Draft	3.95	3,833	99.34	(Zhang et al., 2016)

TABLE S3 The distribution of the COG functional classification in the genome of *A. thiooxidans* ZBY.

COG category	Description	COGs Number	Percent (%)
A	RNA processing and modification	1	0.03
**C**	**Energy production and conversion**	**177**	**4.77**
D	Cell cycle control, cell division, chromosome partitioning	41	1.10
**E**	**Amino acid transport and metabolism**	**145**	**3.91**
F	Nucleotide transport and metabolism	61	1.64
G	Carbohydrate transport and metabolism	97	2.61
H	Coenzyme transport and metabolism	80	2.15
I	Lipid transport and metabolism	61	1.64
J	Translation, ribosomal structure, and biogenesis	139	3.74
K	Transcription	141	3.80
**L**	**Replication, recombination, and repair**	**231**	**6.22**
**M**	**Cell wall/membrane/envelope biogenesis**	**207**	**5.58**
N	Cell motility	54	1.45
O	Posttranslational modification, protein turnover, chaperones	118	3.18
P	Inorganic ion transport and metabolism	127	3.42
Q	Secondary metabolites biosynthesis, transport and catabolism	20	0.54
R	General function prediction only	196	5.28
S	Function unknown	574	15.46
T	Signal transduction mechanisms	113	3.04
U	Intracellular trafficking, secretion, and vesicular transport	104	2.80
V	Defense mechanisms	54	1.45
	No hits	972	26.18

The four most abundant functional categories are shown in bold.

TABLE S4 The fitting results of function F_c (A), F_s (B), and P_s (C) of *A. thiooxidans* pan-genome.

**(a)**				
**ε_c**	**τ_c**	**Ω**	**R-square**	**Adj. R-square[1]**
2,690 ± 421***	2.0 ± 0.54***	1,994 ± 118***	0.9912	0.9883
**(b)**				
**ε_s**	**τ_s**	***tg*(*θ*)**	**R-square**	**Adj. R-square[1]**
93,320 ± 16,520***	0.43 ± 0.04***	28 ± 3***	0.9996	0.9995
**(c)**				
**κ**	**α**		**R-square**	**Adj. R-square[1]**
26,798 ± 7,397***	4.9 ± 0.4***		0.9958	0.9944

The parameter results are in form of estimate ± standard error, and are labeled by '***' when $p < 0.001$.
[1] Adjust R-square = 1-(1-R-square)*(n-1)/(n-k), where n is the number of strains, and k is the number of parameter (here, k=3).

TABLE S5 Classification of core genome of *A. thiooxidans* species using the online platform KAAS.

Pathway Number	Description	CDS Number
**Carbohydrate metabolism**		**172**
ko00010	Glycolysis / Gluconeogenesis	26
ko00020	Citrate cycle (TCA cycle)	14
ko00030	Pentose phosphate pathway	15
ko00040	Pentose and glucuronate interconversions	4
ko00051	Fructose and mannose metabolism	10
ko00052	Galactose metabolism	6
ko00053	Ascorbate and aldarate metabolism	2
ko00500	Starch and sucrose metabolism	10
ko00520	Amino sugar and nucleotide sugar metabolism	20
ko00620	Pyruvate metabolism	22
ko00630	Glyoxylate and dicarboxylate metabolism	21
ko00640	Propanoate metabolism	8
ko00650	Butanoate metabolism	3
ko00660	C5-Branched dibasic acid metabolism	6
ko00562	Inositol phosphate metabolism	5
**Energy metabolism**		**133**
ko00190	Oxidative phosphorylation	50
ko00195	Photosynthesis	8
ko00710	Carbon fixation in photosynthetic organisms	14
ko00720	Carbon fixation pathways in prokaryotes	13
ko00680	Methane metabolism	19
ko00910	Nitrogen metabolism	8
ko00920	Sulfur metabolism	21
**Lipid metabolism**		**40**
ko00061	Fatty acid biosynthesis	9
ko00071	Fatty acid degradation	2
ko00100	Steroid biosynthesis	1
ko00120	Primary bile acid biosynthesis	1
ko00121	Secondary bile acid biosynthesis	1
ko00561	Glycerolipid metabolism	8
ko00564	Glycerophospholipid metabolism	13
ko00565	Ether lipid metabolism	2
ko00600	Sphingolipid metabolism	1
ko00590	Arachidonic acid metabolism	1
ko01040	Biosynthesis of unsaturated fatty acids	1
**Nucleotide metabolism**		**67**
ko00230	Purine metabolism	41
ko00240	Pyrimidine metabolism	26
**Amino acid metabolism**		**142**
ko00250	Alanine, aspartate and glutamate metabolism	13

ko00260	Glycine, serine and threonine metabolism	21
ko00270	Cysteine and methionine metabolism	25
ko00280	Valine, leucine and isoleucine degradation	4
ko00290	Valine, leucine and isoleucine biosynthesis	10
ko00300	Lysine biosynthesis	10
ko00220	Arginine biosynthesis	9
ko00330	Arginine and proline metabolism	11
ko00340	Histidine metabolism	10
ko00350	Tyrosine metabolism	4
ko00360	Phenylalanine metabolism	4
ko00380	Tryptophan metabolism	1
ko00400	Phenylalanine, tyrosine and tryptophan biosynthesis	20
**Metabolism of other amino acids**		**36**
ko00410	beta-Alanine metabolism	4
ko00430	Taurine and hypotaurine metabolism	2
ko00440	Phosphonate and phosphinate metabolism	7
ko00450	Selenocompound metabolism	3
ko00460	Cyanoamino acid metabolism	2
ko00471	D-Glutamine and D-glutamate metabolism	2
ko00473	D-Alanine metabolism	2
ko00480	Glutathione metabolism	14
**Glycan biosynthesis and metabolism**		**41**
ko00531	Glycosaminoglycan degradation	1
ko00540	Lipopolysaccharide biosynthesis	19
ko00550	Peptidoglycan biosynthesis	21
**Metabolism of cofactors and vitamins**		**94**
ko00730	Thiamine metabolism	7
ko00740	Riboflavin metabolism	5
ko00750	Vitamin B6 metabolism	4
ko00760	Nicotinate and nicotinamide metabolism	8
ko00770	Pantothenate and CoA biosynthesis	13
ko00780	Biotin metabolism	9
ko00785	Lipoic acid metabolism	3
ko00790	Folate biosynthesis	15
ko00670	One carbon pool by folate	6
ko00830	Retinol metabolism	1
ko00860	Porphyrin and chlorophyll metabolism	13
ko00130	Ubiquinone and other terpenoid-quinone biosynthesis	10
**Metabolism of terpenoids and polyketides**		**16**
ko00900	Terpenoid backbone biosynthesis	8
ko00909	Sesquiterpenoid and triterpenoid biosynthesis	2
ko00908	Zeatin biosynthesis	1
ko00523	Polyketide sugar unit biosynthesis	4
ko01055	Biosynthesis of vancomycin group antibiotics	1
**Biosynthesis of other secondary metabolites**		**28**
ko00940	Phenylpropanoid biosynthesis	1

ko00950	Isoquinoline alkaloid biosynthesis	1
ko00960	Tropane, piperidine and pyridine alkaloid biosynthesis	3
ko00965	Betalain biosynthesis	1
ko00332	Carbapenem biosynthesis	3
ko00261	Monobactam biosynthesis	4
ko00521	Streptomycin biosynthesis	8
ko00524	Neomycin, kanamycin and gentamicin biosynthesis	1
ko00525	Acarbose and validamycin biosynthesis	2
ko00401	Novobiocin biosynthesis	4
**Xenobiotics biodegradation and metabolism**		**21**
ko00627	Aminobenzoate degradation	2
ko00364	Fluorobenzoate degradation	1
ko00625	Chloroalkane and chloroalkene degradation	1
ko00361	Chlorocyclohexane and chlorobenzene degradation	1
ko00623	Toluene degradation	1
ko00633	Nitrotoluene degradation	2
ko00643	Styrene degradation	1
ko00791	Atrazine degradation	2
ko00626	Naphthalene degradation	1
ko00980	Metabolism of xenobiotics by cytochrome P450	3
ko00982	Drug metabolism - cytochrome P450	3
ko00983	Drug metabolism - other enzymes	3

TABLE S6 The predicted enzymes involved in amino sugar and nucleotide sugar metabolism in all *A. thiooxidans* genomes.

Gene	Description	KO Number	Enzyme Number
nagZ	beta-N-acetylhexosaminidase	K01207	3.2.1.52
amgK	anomeric MurNAc/GlcNAc kinase	K07102	2.7.1.-
murU	MurNAc alpha-1-phosphate uridylyltransferase	K00992	2.7.7.-
glmU	bifunctional UDP-N-acetylglucosamine pyrophosphorylase / Glucosamine-1-phosphate N-acetyltransferase	K04042	2.7.7.23 / 2.3.1.157
murA	UDP-N-acetylglucosamine 1-carboxyvinyltransferase	K00790	2.5.1.7
murB	UDP-N-acetylmuramate dehydrogenase	K00075	1.3.1.98
glmM	phosphoglucosamine mutase	K03431	5.4.2.10
scrK	fructokinase	K00847	2.7.1.4
glk	glucokinase	K00845	2.7.1.2
pgm	phosphoglucomutase	K01835	5.4.2.2
galU	UTP--glucose-1-phosphate uridylyltransferase	K00963	2.7.7.9
ugd	UDPglucose 6-dehydrogenase	K00012	1.1.1.22
galE	UDP-glucose 4-epimerase	K01784	5.1.3.2
	UDP-glucuronate 4-epimerase	K08679	5.1.3.6
manB	phosphomannomutase	K01840	5.4.2.8
rfbA	mannose-1-phosphate guanylyltransferase / mannose-6-phosphate isomerase	K16011	2.7.7.13 / 5.3.1.8
gmd	GDPmannose 4,6-dehydratase	K01711	4.2.1.47
rmd	GDP-4-dehydro-6-deoxy-D-mannose reductase	K15856	1.1.1.281
rfbI	CDP-4-dehydro-6-deoxyglucose reductase, E3	K00523	1.17.1.1
glgC	glucose-1-phosphate adenylyltransferase	K00975	2.7.7.27

TABLE S7 Genes involved in sulfur oxidation and electron transfer in core genome of *A. thiooxidans* species.

**Gene**	**Description**	**KO Number**	**Enzyme Number**
sqr	sulfide quinone oxidoreductase	K17218	1.8.5.4
Tetrathionate hydrolase operon			
tetH	tetrathionate hydrolase		
doxD	thiosulfate:quinone oxidoreductase subunit DoxD	K16936	1.8.5.2
Sulfur-oxidizing protein			
soxA	sulfur-oxidizing protein SoxA	K17222	
soxX	sulfur-oxidizing protein SoxX	K17223	
soxY	sulfur-oxidizing protein SoxY	K17226	
soxZ	sulfur-oxidizing protein SoxZ	K17227	
soxB	sulfur-oxidizing protein SoxB	K17224	
Heterodisulfide reductase			
hdrA	heterodisulfide reductase subunit A		
hdrB	heterodisulfide reductase subunit B		
hdrC	heterodisulfide reductase subunit C		
tst	thiosulfate sulfurtransferase		2.8.1.1
sat	sulfate adenylyltransferase		2.7.7.4
bd ubiquinol oxidases			1.10.3.14
cydA	cytochrome *d* ubiquinol oxidase subunit I	K00425	
cydB	cytochrome *d* ubiquinol oxidase subunit II	K00426	
bo_3 ubiquinol oxidases			1.10.3.10
cyoA	cytochrome o ubiquinol oxidase, subunit II	K02297	
cyoB	cytochrome o ubiquinol oxidase subunit I	K02298	
cyoC	cytochrome o ubiquinol oxidase subunit III	K02299	
cyoD	cytochrome o ubiquinol oxidase subunit IV	K02300	
NADH-ubiquinone oxidoreductase (complex I)			1.6.5.3
nuoA	NADH-ubiquinone oxidoreductase, A subunit	K00330	
nuoB	NADH-ubiquinone oxidoreductase, B subunit	K00331	
nuoC	NADH-ubiquinone oxidoreductase, C subunit	K00332	
nuoD	NADH-ubiquinone oxidoreductase, D subunit	K00333	
nuoE	NADH-ubiquinone oxidoreductase, E subunit	K00334	
nuoF	NADH-ubiquinone oxidoreductase, F subunit	K00335	
nuoG	NADH-ubiquinone oxidoreductase, G subunit	K00336	
nuoH	NADH-ubiquinone oxidoreductase, H subunit	K00337	
nuoI	NADH-ubiquinone oxidoreductase, I subunit	K00338	
nuoJ	NADH-ubiquinone oxidoreductase, J subunit	K00339	
nuoK	NADH-ubiquinone oxidoreductase, K subunit	K00340	
nuoL	NADH-ubiquinone oxidoreductase, L subunit	K00341	
nuoM	NADH-ubiquinone oxidoreductase, M subunit	K00342	
nuoN	NADH-ubiquinone oxidoreductase, N subunit	K00343	

SUPPLEMENTARY REFERENCES

Liljeqvist, M., Valdes, J., Holmes, D. S., and Dopson, M. (2011). Draft genome of the psychrotolerant acidophile *Acidithiobacillus ferrivorans* SS3. *J Bacteriol* 193, 4304-4305.

Talla, E., Hedrich, S., Mangenot, S., Ji, B., Johnson, D. B., Barbe, V. et al. (2014). Insights into the pathways of iron-and sulfur-oxidation, and biofilm formation from the chemolithotrophic acidophile *Acidithiobacillus ferrivorans* CF27. *Res Microbiol* 165, 753-760.

Travisany, D., Cortés, M. P., Latorre, M., Di Genova, A., Budinich, M., Bobadilla-Fazzini, R. A. et al. (2014). A new genome of *Acidithiobacillus thiooxidans* provides insights into adaptation to a bioleaching environment. *Res Microbiol* 165, 743-752.

Valdes, J., Ossandon, F., Quatrini, R., Dopson, M., and Holmes, D. S. (2011). Draft genome sequence of the extremely acidophilic biomining bacterium *Acidithiobacillus thiooxidans* ATCC 19377 provides insights into the evolution of the *Acidithiobacillus* genus. *J Bacteriol* 193, 7003-7004.

Valdés, J., Pedroso, I., Quatrini, R., Dodson, R. J., Tettelin, H., Blake, R. et al. (2008). *Acidithiobacillus ferrooxidans* metabolism: from genome sequence to industrial applications. *BMC Genomics* 9, 597.

Valdes, J., Quatrini, R., Hallberg, K., Dopson, M., Valenzuela, P. D. T., Holmes, D. S. (2009). Draft genome sequence of the extremely acidophilic bacterium *Acidithiobacillus caldus* ATCC 51756 reveals metabolic versatility in the genus *Acidithiobacillus*. *J Bacteriol* 191, 5877-5878.

Yin, H., Zhang, X., Liang, Y., Xiao, Y., Niu, J., Liu, X. (2014). Draft genome sequence of the extremophile *Acidithiobacillus thiooxidans* A01, isolated from the wastewater of a coal dump. *Genome Announc* 2, e00222-14.

You, X. Y., Guo, X., Zheng, H. J., Zhang, M. J., Liu, L. J., Zhu, Y. Q. et al. (2011). Unraveling the *Acidithiobacillus caldus* complete genome and its central metabolisms for carbon assimilation. *J Genet Genomics* 38, 243-252.

Zhang, X., Feng, X., Tao, J., Ma, L., Xiao, Y., Liang, Y. et al. (2016a). Comparative genomics of the extreme acidophile *Acidithiobacillus thiooxidans* reveals intraspecific divergence and niche adaptation. *Int J Mol Sci* 17, 1355.
